# PET VISITATION AS NARRATIVE CARE FOR OLDER ADULTS IN THERAPEUTIC SETTINGS

**DOI:** 10.1093/geroni/igz038.3182

**Published:** 2019-11-08

**Authors:** Jill Yamasaki, Kelley Murfin

**Affiliations:** 1 University of Houston, Houston, Texas, United States; 2 MD Anderson Cancer Center, Houston, Texas, United States

## Abstract

A growing body of research highlights the physiological and psychosocial benefits of pet visitation programs in therapeutic settings. These programs utilize the profound connection between humans and animals to promote holistic healing, foster greater quality of life, and influence meaningful communication between patients and providers. For older adults in hospitals or long-term care, these benefits are often correlated with moments of pleasure, comfort, relaxation, and entertainment. The current study builds on this prior knowledge by examining pet visitation programs as a novel form of narrative care that can also help preserve biographical continuity and promote the sharing of lived stories. We worked with two volunteer pet visitation programs in Houston and one in Los Angeles. Our research included a variety of ethnographic methods, including participant observation; informal interviews with providers, patients (or residents, depending on the context), and their families; semi-structured interviews with volunteers; and discourse review of organizational materials. We employed a method of constant comparison to identify and thematically analyze recurrent patterns of behavior and overarching meanings across the data. Three primary themes emerged from the data: (a) compassion, (b) connection, and (c) response. Collectively, the presence of pets prompted stories and behaviors that foster healing relationships characterized by empathy and mutual understanding between patients (or residents), family members, and providers. Pet visitation programs facilitate storied conversations, increased autonomy, and alternative ways of knowing that promote greater understandings of the patient’s (or resident’s) psychosocial context and biographical history, leading to more personalized care and improved well-being.

